# Evaluating the Capacity of Human Gut Microorganisms to Colonize the Zebrafish Larvae (*Danio rerio*)

**DOI:** 10.3389/fmicb.2018.01032

**Published:** 2018-05-29

**Authors:** Maria-Jose Valenzuela, Mario Caruffo, Yoani Herrera, Daniel A. Medina, Maximo Coronado, Carmen G. Feijóo, Salomé Muñoz, Daniel Garrido, Miriam Troncoso, Guillermo Figueroa, Magaly Toro, Angelica Reyes-Jara, Fabien Magne, Paola Navarrete

**Affiliations:** ^1^Laboratory of Microbiology and Probiotics, Institute of Nutrition and Food Technology (INTA), University of Chile, Santiago, Chile; ^2^Department of Biomedical Engineering, University of Michigan, Ann Arbor, MI, United States; ^3^Department of Chemical and Bioprocess Engineering, School of Engineering, Pontificia Universidad Catolica de Chile, Santiago, Chile; ^4^Departamento de Ciencias Biologicas, Facultad de Ciencias Biologicas, Universidad Andres Bello, Santiago, Chile; ^5^Departamento de Biología, Facultad de Ciencias, Universidad de Chile, Santiago, Chile; ^6^Microbiology and Mycology Program, Faculty of Medicine, Institute of Biomedical Sciences, University of Chile, Santiago, Chile

**Keywords:** zebrafish, human microbiota, humanization, *Clostridioides difficile*, *Bifidobacterium*, *Lactobacillus*

## Abstract

In this study we evaluated if zebrafish larvae can be colonized by human gut microorganisms. We tested two strategies: (1) through transplantation of a human fecal microbiota and (2) by successively transplanting aerotolerant anaerobic microorganisms, similar to the colonization in the human intestine during early life. We used conventionally raised zebrafish larvae harboring their own aerobic microbiota to improve the colonization of anaerobic microorganisms. The results showed with the fecal transplant, that some members of the human gut microbiota were transferred to larvae. *Bacillus, Roseburia, Prevotella, Oscillospira*, one unclassified genus of the family Ruminococcaceae and Enterobacteriaceae were detected in 3 days post fertilization (dpf) larvae; however only *Bacillus* persisted to 7 dpf. Successive inoculation of *Lactobacillus, Bifidobacterium* and *Clostridioides* did not improve their colonization, compared to individual inoculation of each bacterial species. Interestingly, the sporulating bacteria *Bacillus clausii* and *Clostridioides difficile* were the most persistent microorganisms. Their endospores persisted at least 5 days after inoculating 3 dpf larvae. However, when 5 dpf larvae were inoculated, the proportion of vegetative cells in larvae increased, revealing proliferation of the inoculated bacteria and better colonization of the host. In conclusion, these results suggest that it is feasible to colonize zebrafish larvae with some human bacteria, such as *C. difficile* and *Bacillus* and open an interesting area to study interactions between these microorganisms and the host.

## Introduction

Microorganism-host interactions can be explored using traditional experimental approaches such as *in vitro* experiments through the use of different cell lines. However, these fail to reproduce well the biological context shaped by the host. Animal models such as germ-free or gnotobiotic mice are costly and labor intensive. In addition, there are major ethical concerns associated with mammal models. For these reasons, over the last few years new simple biological non-mammalian models such as zebrafish have been developed. The zebrafish have several experimental and developmental advantages that reinforce their utility as a model organism: (i) they are easy to maintain and breed, (ii) they develop very rapidly with short generation time, (iii) they can produce a large number of offspring, (iv) the transparency of the early larval stages allows easy observation of internal organs and the observation of colonizing microorganisms, (v) availability of transgenic lines expressing fluorescent proteins in different cell lineages permits high-resolution *in vivo* observation of physiological and physio-pathological processes, (vi) the zebrafish genome has been sequenced, allowing the study of the expression of molecular determinants participating in fish physiology, and (vii) methodologies for rearing germ-free and gnotobiotic zebrafish have been standardized (Pham et al., 2008).

The zebrafish digestive tract shares extensive homology with that of mammals, including liver, gall bladder, endocrine and exocrine pancreas, and an intestine with proximal-distal functional specification. Initial morphogenesis is completed by 3 days post-fertilization (dpf), and the intestine can be colonized by external microorganisms at this stage (Rawls et al., 2004; Bates et al., 2006). Interestingly, the role of the gut microbiota on host biology is similar between zebrafish and mammals. Both microbiota participate in the education of the immune system, maturation of the gut and promotion of nutrient metabolism in the host (Rawls et al., 2004, 2007; Bates et al., 2006, 2007). Thus the zebrafish is suggested as an interesting vertebrate model to study the interaction between the host and human gut commensal microbiota or pathogenic microorganisms (Kanther and Rawls, 2010; Milligan-Myhre et al., 2011). A few studies have shown that the gastrointestinal tract of zebrafish can support the transplantation of mouse-associated microbiota (Rawls et al., 2006) and some human microbiota isolates (Toh et al., 2013). However, more studies are needed to confirm the use of this model to apply it to human gut microbiota-host interactions.

While the intestine of zebrafish larvae is predicted to harbor higher oxygen concentration than the mammalian intestine (Rawls et al., 2006), it has been shown that two anaerobic bacteria of the commensal human microbiota can survive in the zebrafish larval gut (Toh et al., 2013). In this study, larvae were inoculated by immersion with a defined community of 30 nonpathogenic, obligate anaerobic microorganisms of which *Eubacterium limosum* and *Lactobacillus paracasei*, representing about 7% of the inoculated microorganisms, could colonize the gut of germ-free zebrafish larvae from 5 to 12 days post-fertilization (dpf) (Toh et al., 2013). This suggests that is feasible to introduce specific human gut microbiota to the digestive tract of the zebrafish. However, to date it is not known whether zebrafish larvae can support a human fecal transplantation. In addition, little knowledge exists about the factors involved in their colonization capacity and persistence in the gut.

In this study we evaluated two factors to improve the colonization and increase the persistence of human gut microorganisms in the zebrafish intestine: (1) maintaining the interaction between microorganisms, through the transplantation of the complete human microbiota (not just some bacterial components such as in Toh et al., 2013), and (2) the timing of the inoculation, inoculating successively different human aerotolerant anaerobic microorganisms which have been frequently identified in the infant gut microbiota, such as *Lactobacillus, Bifidobacterium* and *C. difficile* (Penders et al., 2006; Houghteling and Walker, 2015). It is known that the early colonization of the microbiota in human neonates starts with aerobic microorganisms that consume the oxygen present in the intestine and then favors the implantation of anaerobic bacteria (El Aidy et al., 2013). Therefore, we performed the experiments on conventionally raised larvae harboring their own gut microbiota which are known to be aerobic, in order to facilitate the implantation of less aerotolerant or anaerobic human microorganisms.

## Materials and methods

### Bacterial strains, zebrafish, and culture conditions

*Lactobacillus acidophilus* (ATCC®4356™), *Bifidobacterium adolescentis* (ATCC®15703™) and *Clostridioides difficile* (N339), all of human origin, were cultured in MRS (Difco), MRS supplemented with 0.05% L-cysteine and NN (40 g/L Bacto peptone, 5 g/L Na_2_HPO_4_, 1 g/L KH_2_PO_4_, 2 g/L NaCl, 0.1 g/L MgSO_4_, 2 g/L glucose, 25 g/L Bacto agar and 10% v/v egg yolk), respectively, and incubated at 37°C for 48 h in anaerobic jars with anaerobic sachets (GasPak^TM^EZ, BD). The strain N339 was isolated from a fecal sample of a healthy infant by culture in NN agar and incubation in an anaerobic jar (Brunser et al., 2006) and phenotypically identified as *Clostridium* sp. This strain was chosen from 20 isolates of our *Clostridium* collection because of its aerotolerance and ability to be easily cultured in an anaerobic jar. In this study, this strain was identified as *Clostridium difficile* according to its 16S rRNA sequencing (KY523549). However, it was proposed in 2016 that *Clostridium difficile* should be reclassified as *Clostridioides difficile* (Lawson et al., 2016). We also included the commercial probiotic strain *Bacillus clausii* (Enterogermina®) of unknown origin (Green et al., 1999), which was grown in Tryptic Soy Agar (TSA, Difco; 15 g/L pancreatic digest of casein, 5 g/L papaic digest of soybean, 5 g/L NaCl, 15 g/L agar) at 37°C for 24 h. After growing, an isolated colony was twice purified and stored at −80°C until used. Gram (Gram staining kit; ChemiX) and green malachite (Merck) staining were performed according to manufacturer instructions.

Tab5 zebrafish embryos (*Danio rerio*) were collected by natural spawning staged according to a previous report (Kimmel et al., 1995) and raised as reported (Hedrera et al., 2013) at 28°C in sterile E3 medium (1% NaCl, 0.17 mM KCl, 0.33 mM CaCl_2_, 0.33 mM MgSO_4_ and 0.00003% methylene blue, pH7.0) in sterile Petri dishes (50 embryos/dish). 75% of the E3 volume was replaced daily with sterile E3 to avoid waste accumulation and oxygen limitation. At 3 dpf, larvae were transferred to six-well sterile culture plates (20 larvae/well). When necessary, larvae were euthanized with an overdose of tricaine methane sulfonate (4%, MS-222, Sigma-Aldrich). All zebrafish husbandry and experimental procedures were performed in accordance with relevant guidelines and regulations and approved by the Committee on the Ethics of Animal Experiments of INTA, University of Chile.

### Zebrafish inoculation with a human gut microbiota and bacterial isolates

Zebrafish larvae were inoculated with fecal microbiota of a normal weight (BMI < 22) 35-year old donor. The protocol for the fecal sampling from the healthy volunteer and experimental procedure was performed in accordance with relevant guidelines and regulations and approved by the Institutional Review Board of INTA, University of Chile, together with written informed consent of the volunteer. The fecal sample was transported to the laboratory in anaerobiosis and processed within 2 h after collection, as previously reported (Goodman et al., 2011). In brief, 3 g of fecal sample was homogenized for 5 min with 45 mL of pre-reduced sterile PBS with 0.1% cysteine and allowed to stand for 5 min. 100 zebrafish larvae (3 dpf) were inoculated, in duplicate (inoculated larvae 1 and 2), with a 1/10 dilution of the supernatant (fecal inoculum), in sterile E3 medium. After 30 min, the bacterial inoculum was removed and in aerobic and sterile conditions larvae were washed at least 4 times with sterile E3 and then maintained with sterile E3 at 28°C. After 2 h, larvae were washed with sterile E3 and sampled as described below. In colonization studies with human bacterial isolates, 60 zebrafish larvae (3, 4, or 5 dpf) were inoculated by immersion with a suspension of each bacterium at a final concentration of 10^8^ CFU/ml in sterile E3 medium, as described above. Colonization experiments with bacterial strains were performed 3 times. To evaluate bacterial colonization, the microbiota of the inoculated and non-inoculated larvae were identified from 3 to 7 or 9 dpf larvae with molecular and culture methods, as described below.

### Identification of the microbiota

For the identification of the human gut microbiota by culture, serial dilutions of the fecal supernatant (obtained as described above) in pre-reduced PBS were plated in M2GSC (0.09% (NH4)_2_SO_4_, 0.009% CaCl_2_, 1% casitone, 0.2% cellobiose, 30% v/v clarified rumen fluid, 0.1% cysteine, 0.2% glucose, 0.045% K_2_HPO_4_, 0.045% KH_2_PO_4_, 0.009% MgSO_4_ x 7 H_2_O, 0.09% NaCl, 0.2% NaHCO_3_, 0.2% soluble starch, 0.25% yeast extract), NN, MRS, and MRS-0.05% cysteine media and incubated in an anaerobic jar for 4 days at 37°C. In addition, they were plated in TSA medium which was incubated for 4 days at 37° and 28°C. All different isolated colonies were purified and identified by sequencing their 16S rRNA gene, as described below. Colonies from NN and M2GSC medium containing more than 1000 colonies were harvested by scraping and their DNA extracted for bacterial identification by MiSeq sequencing of the 16S rRNA gene. In addition, the human gut microbiota was identified by a molecular method (MiSeq sequencing of the 16S rRNA gene). To check that the bacterial diversity of the fecal inoculum for larvae was similar to the solid fecal sample of the human volunteer, molecular analysis was performed in these two samples. As described above, the fecal inoculum for larvae corresponds to the supernatant obtained after homogenization of the solid fecal sample for 5 min with 45 mL of pre-reduced sterile PBS with 0.1% cysteine and allowed to stand for 5 min. Therefore, DNA was extracted from 200 mg of the solid fecal sample and for the fecal inoculum, DNA was extracted from the pellet obtained after centrifuging 1 mL of the supernatant at 10,000 g for 5 min.

To evaluate the bacterial microbiota of inoculated and non-inoculated larvae, 6 larvae of each group were sampled daily, euthanized via a tricaine overdose, washed and homogenized with sterile PBS and plated in the respective medium and incubated as described above. Homogenization of larvae and inoculation into agar media was performed in aerobic conditions. In addition, for the microbiota identification of the larvae with MiSeq sequencing, DNA was extracted from the inoculated and non-inoculated zebrafish larvae (70 larvae homogenized in sterile E3) as described below.

### DNA extraction, 16s rRNA sequencing, and sequence treatment

DNA was directly extracted from samples with the QIAmp DNA Stool Mini Kit (Qiagen, Hilden, Germany) with a previous bead-beater treatment of 5 min (Morales et al., 2016). Libraries and sequencing were performed as previously described (Morales et al., 2016) in the Roy J. Carver Biotechnology Center at the University of Illinois at Urbana-Champaign, Champaign, IL, USA. The V3–V4 region of the 16S rRNA gene was amplified with the primers 341F (5′-CCTACGGGNGGCWGCAG-3′) and 785R (GACTACHVGGGTATCTAATCC-3′) using the Fluidigm system (Fluidigm, South San Francisco, CA). Amplicons were sequenced on a MiSeq Illumina platform (Illumina, San Diego, CA), generating paired-end reads (2 × 300 nt). Demultiplexes and barcode-depleted sequences were delivered from sequencing services (W.M. Keck Center for Comparative and Functional Genomics, University of Illinois, USA). Paired-end sequences were joined using PEAR (Zhang et al., 2014) and primer sequences were depleted by fastx-trimmer from FASTX-Toolkit. The Quantitative Insights Into Microbial Ecology (QIIME, v1.9.1) software was used to analyze the 16S rRNA gene sequences (Caporaso et al., 2010; Navas-Molina et al., 2013). Operational taxonomic units (OTUs) were picked by open-reference command and defined by clustering at 3% divergence (97% similarity) using as reference the GreenGenes database (DeSantis et al., 2006; McDonald et al., 2012) release 08-2013. Negative control OTUs were removed from all samples.

To determine bacterial composition using culture, DNA from each bacterial isolate was extracted using the Wizard® genomic kit (Promega, Madison, WI, USA). Amplification of the 16S rRNA gene was performed with the primers 27F 5′- AGAGTTTGATCCTGGCTCAG-3′ and 1492R 5′-ATTTGCTAAAGCGGGAATCT-3′ as previously described (Romero and Navarrete, 2006). PCR reactions were performed in a reaction mixture containing 1X GoTaq®Green master Mix (Promega, Madison, WI, USA) and 0.25 pmol μl^−1^ of each primer. The PCR consisted of an initial denaturation time of 3 min at 95°C followed by 30 cycles of amplification consisting a denaturation step for 1 min 30 s at 95°C, annealing at 58°C for 1 min 30 s and extension at 72°C for 1 min 30 s. Reactions were completed with 10 min elongation at 72°C followed by cooling to 4°C. PCR products were purified and sequenced by the Macrogen USA sequencing service. Sequences were edited and cleaned using BIOEDIT software (http://www.mbio.ncsu.edu/BioEdit/bioedit.html). Each sequence was analyzed to find GenBank sequences with close BLAST-N hits. Similarities between sequences were assessed using pairwise distance calculation.

### Bacterial localization in zebrafish larvae

The *in vivo* localization of *L. acidophilus* (ATCC®4356™), *B. adolescentis* (ATCC®15703™), *Clostridioides difficile* (N339) and *Bacillus clausii* in 5 dpf zebrafish larvae was determined through the inoculation of fluorescent bacteria labeled with 5-([4,6-Dichlorotriazin-2-yl] amino) fluorescein hydrochloride (DTAF, Sigma–Aldrich) and immersed in E3 at a concentration of 10^8^ CFU/ml as previously reported (Caruffo et al., 2015; *n* = 60 larvae per bacterium). Because we did not know if DTAF could affect bacterial replication or if the progeny could retain the fluorescence, this experiment was performed to observe the *in vivo* localization of bacteria in larvae after 2 h of being inoculated and to confirm that larvae ingested the bacteria and not used to evaluate the persistence capacity of the inoculated strains, which was determined by culture (Figures 5, 6). Observation of larvae was performed in an SZX16 stereoscope (Olympus) with a Micro Publisher 5.0 RVT camera (QImaging) and a confocal microscope Olympus FluoView FV1000 Spectral, Software version 2.1. Each experiment was performed independently three times.

### Survival of bacterial isolates in E3 medium

The survival of *L. acidophilus* (ATCC®4356™), *B. adolescentis* (ATCC®15703™), *Clostridioides difficile* (N339), and *B. clausii* was determined in the E3 zebrafish medium at a concentration of 10^8^-10^9^ CFU/ml. Inoculated medium was incubated at 28°C in aerobiosis. Counts of viable bacteria were made daily by plating on MRS, MRS supplemented with 0.05% L-cysteine, NN, and TSA respectively, and incubated as described above. Each experiment was performed independently three times.

### Determination of endospores and vegetative cells in zebrafish larvae and E3 medium

To determine the concentration of endospores colonizing the larvae, similar colonization experiments were performed as described above, but each day homogenized larvae were treated at (i) 75°C for 15 min, (ii) lysozyme (2 mg/mL) for 1 h at 37°C, and (iii) 50% ethanol for 30 min at 37°C (Barra-Carrasco et al., 2013; Edwards et al., 2016), as previously reported with slight modifications. After treatments, ten-fold dilutions were plated in duplicate in NN and TSA medium, incubated in anaerobiosis and aerobiosis at 37°C for 48 h to count endospores of *C. difficile* and *B. clausii*, respectively. To count the endospores in E3 medium, inoculated E3 was treated daily similarly to the homogenized larvae. Total bacterial counts were determined as described above.

### Statistical analysis

Alpha and beta diversity metrics were calculated using the scripts provided by QIIME v1.9.1 (Caporaso et al., 2010; Navas-Molina et al., 2013). Alpha diversity was calculated using Phylogenetic Distance over OTU counts (Faith, 1992). The differences in diversities among samples were estimated by beta diversity using the Unifrac metric (Lozupone et al., 2006). The Unifrac values obtained were used as a dissimilarity metric between samples to build PCoA plots (Mardia et al., 1979). To visualize the abundance of taxa in each sample, OTUs were grouped according to taxonomic level and relative abundance was represented as percentage in bar plots, where color height represents the percentage contribution of each taxon group (Navas-Molina et al., 2013). To ensure that the depth of DNA sequencing described most of the diversity in the samples studied, we plotted rarefaction curves with the number of observed OTUs vs. number of sequence for each sample (Kuczynski et al., 2012). Statistical analysis was performed using the GraphPad Prism 6 software (Graphpad Software, Inc.). Differences in mean concentration of bacteria were analyzed by Mann-Whitney test, and experiments were performed at least three times. *P* ≤ 0.05 was considered significant.

## Results

**Figure 1:**
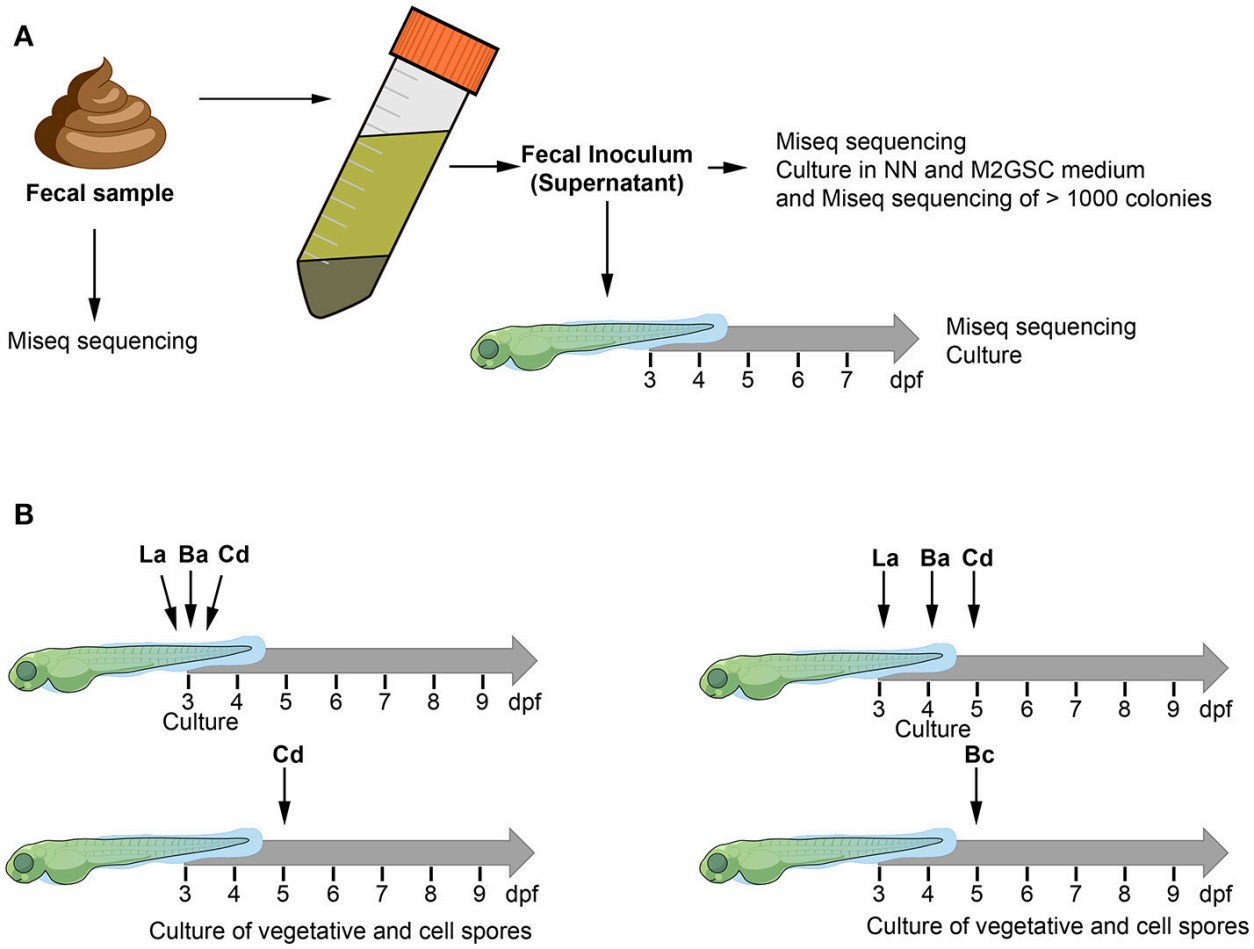
The figure illustrates the two experimental approaches used to humanize the zebrafish larvae. The composition of bacterial microbiota was analyzed through culture and MiSeq sequencing **(A)** Fecal sample was homogenized with pre-reduced PBS (0.1% cysteine) and allowed to stand for 5 min. The supernatant of the homogenized solution was used as inoculum to transplant 3 dpf zebrafish larvae. **(B)** Zebrafish larvae (3, 4, or 5 dpf) were inoculated by immersion with a suspension of each bacterium at a final concentration of 10^8^ CFU/mL in sterile E3 medium. La: *Lactobacillus acidophilus*, Ba: *Bifidobacterium adolescentis*, Cd: *Clostridioides difficile*, Bc: *Bacillus clausii*.

We tested two experimental strategies to humanize the zebrafish with human gut microbiota (Figure 1). We first hypothesized that the existing interactions between the microorganisms of the microbiota can improve the colonization of human microorganisms in zebrafish larvae, thus we first inoculated a fecal sample (human fecal transplantation). The fecal sample was homogenized according to previous recommendations (Goodman et al., 2011) to prepare the fecal inoculum, which was transplanted by immersion into the larval medium (sterile E3) of 3 days post fertilization (dpf) larvae (Figure 1A). The composition of the microbiota of the control non-inoculated larvae, the fecal sample, the fecal inoculum, and the inoculated larvae were identified through the sequencing of the 16S rRNA gene using MiSeq (Illumina) and culture (Figure 1A).

**Figure 2:**
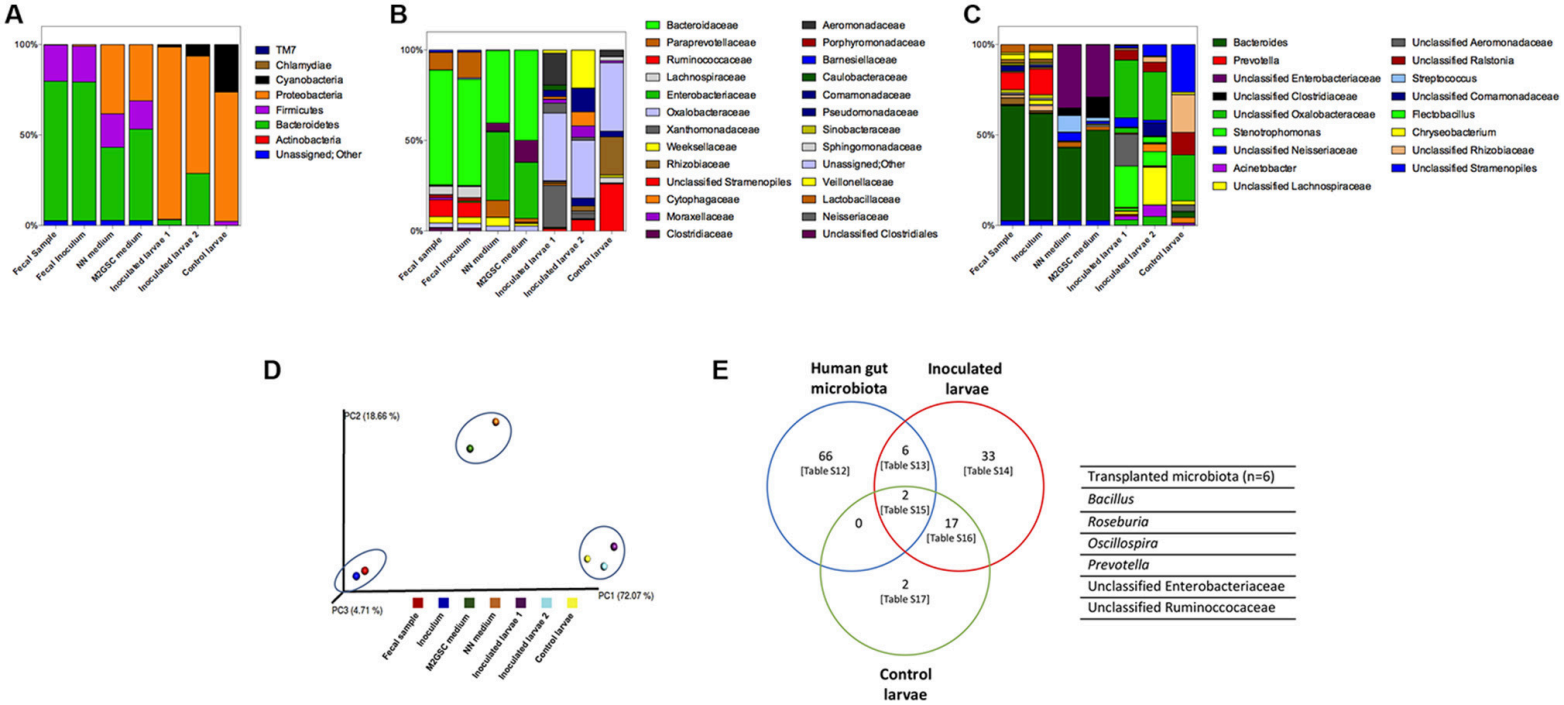
Composition of the bacterial microbiota of samples through 16S rRNA sequencing by MiSeq. Relative abundance of **(A)** phyla, **(B)** families (legend shows families with relative abundance greater than 1%), and **(C)** genera (legend shows genera with relative abundance greater than 5%). The complete description of the family and genus legends is given in the Supplementary Material (Figures S2A,B). Samples analyzed were fecal sample, inoculum of the fecal sample, >1,000 colonies recovered on NN and M2GSC media, inoculated larvae with fecal inoculum (in duplicate: inoculated larvae 1 and 2), and non-inoculated control larvae. **(D)** Principal Coordinate Analysis (PCoA) scores plot based on the relative abundance of OTUs (97% similarity level). Each colored symbol represents a sample. Blue circles grouped similar samples based on their bacterial composition. Inoculated larvae corresponded to 3 dpf larvae after 2.5 h of been inoculated with the human gut inoculum. The persistence of these transplanted human fecal microorganisms was evaluated in larvae from 4 to 7 dpf through direct sequencing of the 16S rRNA gene by MiSeq, however it was not possible to amplify bacterial DNA from these samples and persistence were checked by culture (Table 1). **(E)** Human gut microbiota transplanted to 3 dpf zebrafish larvae (the names of the transplanted species are detailed in the right side of the Venn diagram; Table S13). Tables S12–S17 are detailed in Supplementary Material.

The MiSeq sequencing revealed a total of 467,275 sequences after trimming, assembly, quality filtering and chimera checking, with an average sequence length of 440.7 ± 9.8 bp. Sequences were grouped using open-reference OTU picking and clustering at 97% similarity, using the GreenGenes 13_8 database as reference. After subtracting the counts belonging to the negative control (to eliminate possible background contamination from reagents), a total of 285,312 counts were obtained in a BIOM table, with an average of 40,758 ± 28,241 counts per sample (ranging from 16,221 to 107,358). OTUs with at least 2 observation counts were considered for further analysis; they were classified into 7 phyla, 63 families and 119 genera (Figures 2A–C; Table S1). The rarefaction curves tended to the saturation plateau for each sample (Figure S1).

### The gut microbiota of conventionally raised larvae (control non-inoculated larvae)

The bacterial microorganisms of the developing larvae were identified from 3 to 7 dpf using culture and 16S rRNA gene sequencing (MiSeq) of the total DNA extracted directly from the 3 dpf larvae. The massive sequencing of the gut microbiota of 3 dpf larvae revealed that Proteobacteria (71%) and Cyanobacteria (26%) were the predominant phyla, whereas TM7 accounted for 0.01% (Figure 2A). The most abundant families were Oxalobacteraceae (38%), an unclassified family of the Cyanobacteria (26%) and Rhizobiaceae (21%) (Figure 2B, Table S1). The most abundant genera were one taxon of Cyanobacteria (26%), one of the family Rhizobiaceae (Alphaproteobacteria) (20%), one of the family Oxalobactereraceae (Betaproteobacteria) (25%) and *Ralstonia* (12%). *Pseudomonas, Stenotrophomonas, Acinetobacter, Aeromonas, Chryseobacterium, Rhizobium, Sphingomonas, Acidovorax* and *Comamonas* were detected in lower abundance (Figure 2C, Table S1). Culture analysis showed that *Pseudomonas* and *Stenotrophomonas* were present at 3 dpf and *Acinetobacter, Aeromonas jandaei, Chryseobacterium*, and *Herbaspirillum huttiense* between 3 and 7 dpf (Table 1).

**Table 1:** Bacterial genera identified by culture analysis and sequencing of their 16S rRNA gene from fecal samples and zebrafish larvae.

	**Fecal**	**Fecal**	**Inoculated larvae (dpf)**					**Control larvae (dpf)**				
**Bacterial genus**	**Sample**	**Inoculum**	**3**	**4**	**5**	**6**	**7**	**3**	**4**	**5**	**6**	**7**
*Bifidobacterium*	•	•										
*Lactobacillus*	•											
*Escherichia coli/ Shigella*	•	•										
*Enterococcus faecium*	•	•										
*Streptococcus salivarius*	•											
*Bacillus*	•		•		•							
*Herbaspirillum huttiense*		•		•	•	•	•	•	•	•	•	•
												
*Ralstonia pickettii*			•									
*Aerococcus*			•									
*Pseudomonas*			•					•				
*Stenotrophomonas*			•	•	•	•	•	•				
*Acinetobacter*				•	•	•	•	•	•	•	•	•
*Aeromonas jandaei*						•		•	•	•	•	•
*Chryseobacterium*								•	•	•	•	•

*All samples were plated in TSA, MRS, MRS-cysteine, NN and M2GSC media and incubated at 37° and 30°C. Isolated colonies were purified twice in the same medium and identified through sequencing their 16S rRNA gene*.

### Analysis of the gut microbiota of a human volunteer (fecal sample and inoculum)

In the human fecal sample we identified two abundant phyla, Bacteroidetes (77.1%) and Firmicutes (20.0%), and less abundant phyla Proteobacteria and Actinobacteria accounting for < 1%, and 0.2% corresponding to unassigned sequences (Figure 2, Table S1). The most abundant families were Bacteroidaceae (63%), Ruminococcaceae (9%), Lachnospiraceae (5%) and Veillonellaceae (4%), with Bacteroides (63%), one genus of the family Ruminococcaceae (4%) and Lachnospiraceae (3%), *Dialister* (3%), *Ruminococcus* (3%), and *Faecalibacterium* (1%) as abundant genera. The homogenization of the sample was performed as previously reported (Goodman et al., 2011), to preserve the majority of the anaerobic microbiota. In brief, 3 g of fecal sample was homogenized for 5 min with 45 mL of sterile PBS with 0.1% cysteine and allowed to stand for 5 min. The supernatant was used as inoculum to colonize larvae. The bacterial composition of this inoculum was similar to the fecal sample (when both samples were analyzed with MiSeq sequencing (Figure 2, Tables S2–S4, Figure S1) and grouped together in the PCA analysis (Figure 2D).

The bacterial composition of the inoculum was also analyzed by culture (in NN and M2GSC media). We harvested the total colonies (>1,000) present in NN and M2GSC media, and identified the microorganisms by sequencing the 16S rRNA gene using MiSeq. Interestingly, the data revealed that it was possible to culture some fastidious microorganisms such as *Veillonella*, different microorganisms belonging to the family *Lachnospiraceae, Oscillospira, Faecalibacterium*, unclassified Clostridiaceae, *Eggerthella* and *Phascolartobacterium*. The two methods (direct MiSeq sequencing and culture and sequencing) were complementary, since each identified some exclusive bacterial taxa (Tables S5–S11, Figure S1C).

### Characterization of gut microbiota in zebrafish with transplanted human feces (inoculated larvae)

We evaluated the capacity of the human gut microbiota to colonize the zebrafish larvae through fecal microbiota transplantation. Of the 74 genera identified in the fecal sample, 6 were detected in larvae at 3 dpf: *Bacillus, Roseburia, Oscillospira, Prevotella*, one unclassified genus of the family Ruminococcaceae and Enterobacteriaceae (Figure 2E). The transfer of the 6 bacterial taxa from the fecal inoculum to inoculated larvae was not sufficient to modify their microbiota and to ungroup these samples from non-inoculated larvae (control larvae) on the Principal Coordinate Analysis plot (Figure 2D). The persistence of these transplanted human fecal microorganisms was evaluated in larvae from 4 to 7 dpf through direct sequencing of the 16S rRNA gene by MiSeq, and by culture. Unfortunately, it was not possible to amplify bacterial DNA from larvae; therefore the persistence was checked by culture (Table 1). Only *Bacillus* was detected at days 3 and 5 dpf in the developing larvae. *Roseburia, Oscillospira, Prevotella*, one unclassified genus of the family Ruminococcaceae and Enterobacteriaceae were not detected from 4 to 7 dpf larvae, perhaps because they could not persist in larvae or their concentrations were below the detection limit of detection methods used. Future experiments will be performed to assess their presence with more sensitive methods such as quantitative PCR.

### Human bacterial strains inoculated into zebrafish larvae are located in the gut

**Figure 3:**
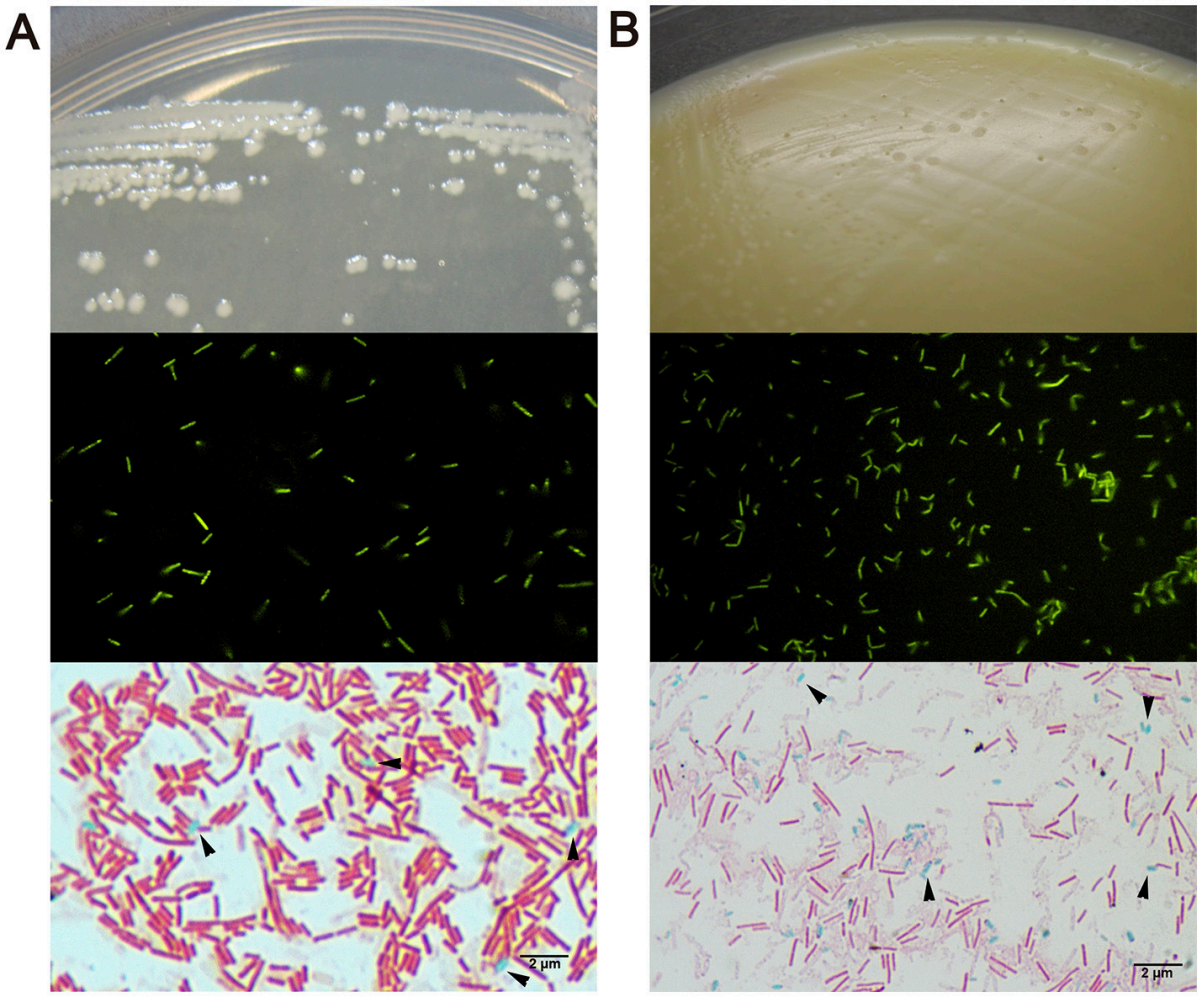
Macroscopic and microscopic morphology of vegetative cells and endospores of **(A)** *Bacillus clausii* and **(B)** *Clostridioides difficile*. Upper show bacterial colonies in TSA or NN media. Middle show DTAF fluorescent bacteria. Lower show green malachite staining with black arrows indicating green endospores.

**Figure 4:**
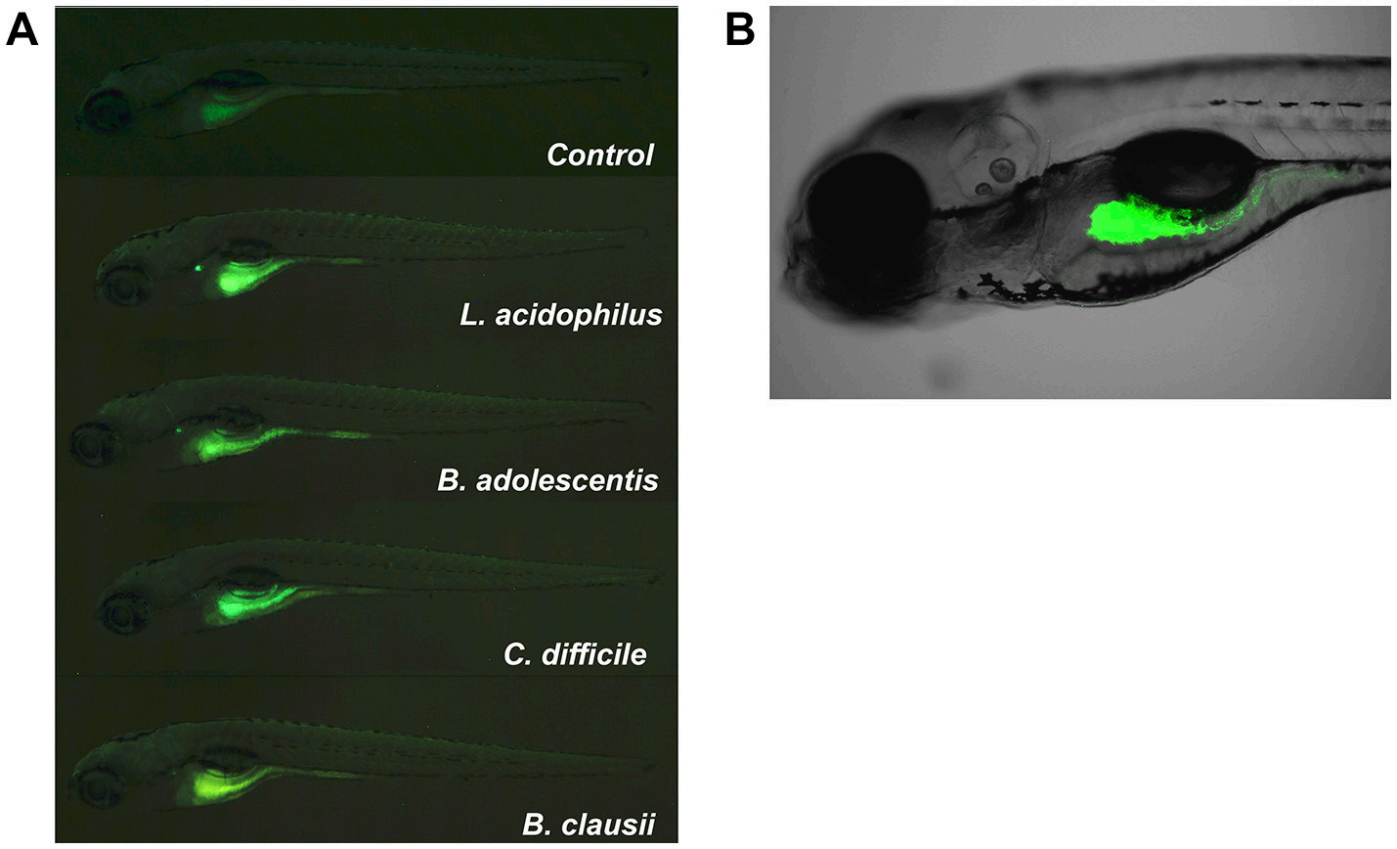
Localization of fluorescent bacteria (DTAF) in 5 dpf zebrafish larvae. Observation of larvae in an **(A)** SZX16 stereoscope (Olympus) with a Micro Publisher 5.0 RVT camera (QImaging) and **(B)** confocal laser scanning microscope Olympus FluoView FV1000 Spectral, Software version 2.1. The figure shows different larvae after 2 h of being inoculated with DTAF-labeled bacteria.

We then evaluated a second approach to colonize zebrafish larvae by introducing different bacterial strains of human origin (*L. acidophilus, B. adolescentis, Clostridioides difficile*), corresponding to bacterial microorganisms highly prevalent in the gut microbiota of infants (Penders et al., 2006; Houghteling and Walker, 2015). These bacteria are aerotolerant anaerobic microorganisms, which could facilitate their survival in the aerobic larval gut. We first localized the strains in zebrafish larvae. We labeled each bacteria with DTAF and fluorescent bacteria (Figure 3, middle panels) were inoculated by immersion in the zebrafish medium (E3) of 5 dpf larvae. The transparency of larvae at this stage was utilized to observe the localization of fluorescent bacteria using a stereomicroscope and confocal microscope. *L. acidophilus, B. adolescentis* and *C. difficile* were all located in the gastrointestinal tract of larvae (Figure 4 and Supplementary Video 1).

### *Clostridioides difficile* was the most persistent strain in the zebrafish larvae

We further estimated the colonization capacity of these human bacterial strains by counting the survival bacteria in larvae (CFU/ larvae). First, *L. acidophilus, B. adolescentis*, and *C. difficile* were sequentially inoculated at 3 dpf (Figure 1B), or sequentially inoculated on days 3 (*L. acidophilus*), 4 (*B. adolescentis*), and 5 (*C. difficile*) dpf (Figure 1B). Larvae were inoculated by immersion with a suspension of each bacterium at a final concentration of 10^8^ CFU/ml in sterile E3 medium. In the case of *C. difficile*, the suspension was performed from a sporulating culture obtained by growing the bacteria 48 h in NN medium in an anaerobic jar. This sporulating culture (total cell count) contained a vegetative/endospore proportion of about 90/10%, as observed by microscopy (Figure 3B, lower panel). Colonization analysis of *C. difficile* in larvae was first achieved by counting the total cells (vegetative and endospore cells) per larvae.

**Figure 5:**
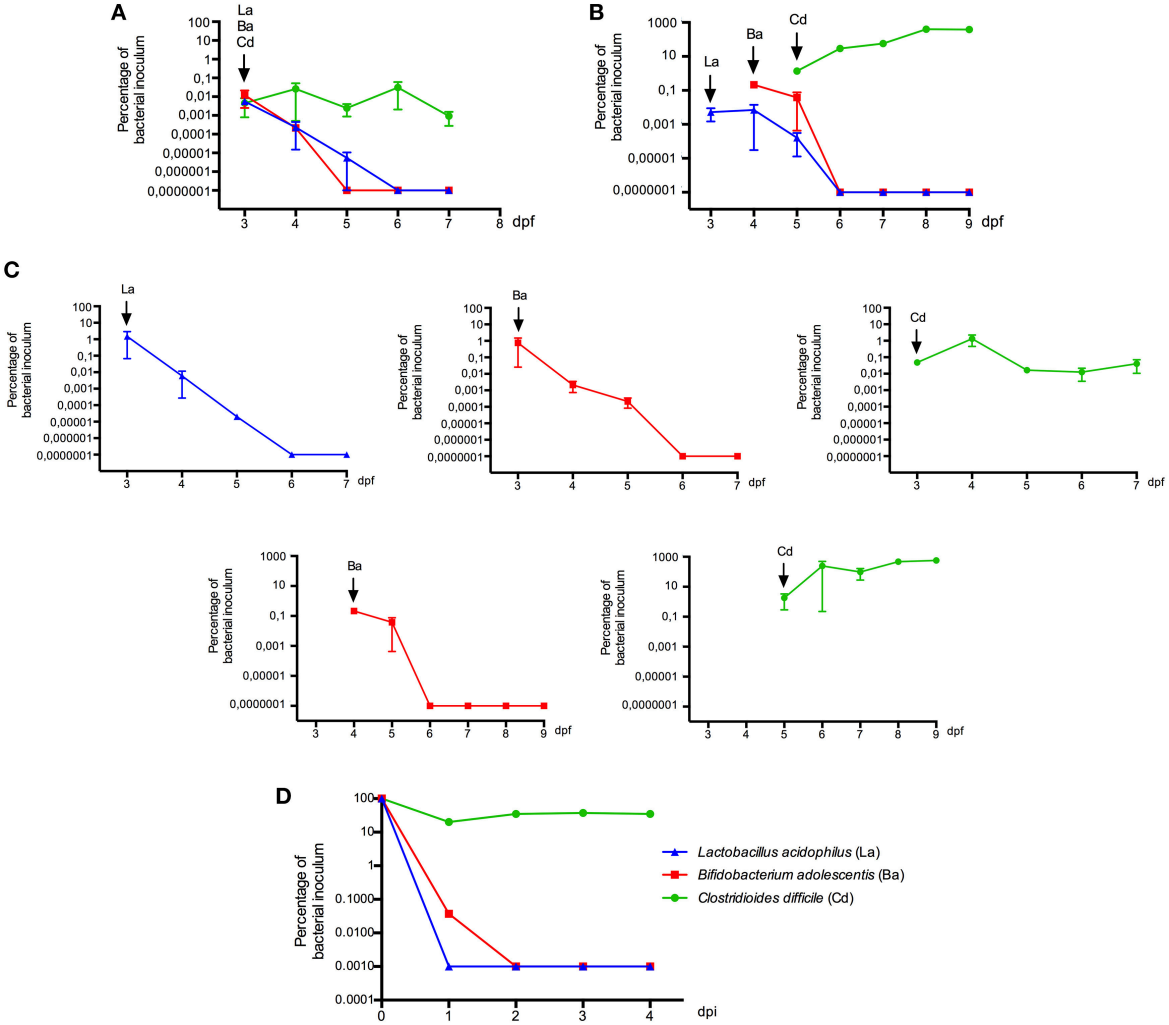
Colonization of zebrafish larvae (3, 4, or 5 dpf) with human bacterial isolates. Bacterial strains were successively inoculated by immersion with a suspension of each bacterium at a final concentration of 10^8^ CFU/ml in sterile E3 medium **(A)** on day 3 dpf, **(B)** on day 3, 4, and 5 dpf. **(C)** Colonization of bacterial strains when they were inoculated alone. Bacterial colonization was determined by culture. **(D)** Viability of each bacterial strain in E3 medium. La: *Lactobacillus acidophilus*, Ba: *Bifidobacterium adolescentis*, Cd: *Clostridioides difficile*, Bc: *Bacillus clausii*. Each colonization experiment was performed independently at least 3 times; the results show mean ± SE and Mann-Whitney test performed.

We observed that *L. acidophilus* and *B. adolescentis* can persist until 2 days after inoculation. *C. difficile* persisted with the same concentration in larvae until the end of the experiment (P = 0.0857, Mann Whitney) when it was inoculated on 3 dpf larvae (Figure 5A). However, *C. difficile* proliferated when inoculated on 5 dpf larvae, reaching almost 3 log higher at day 9 dpf than on the day of inoculation (*P* = 0.0002, Mann Whitney; Figure 5B). We could not detect any positive or negative interaction between the inoculated strains when they were inoculated together in the same larvae, since colonization of single strains displayed similar patterns (Figure 5C).

In order to evaluate some interactions of these strains with the host, we compared their viability in larvae and in E3 medium. The survival of *L. acidophilus* and *B. adolescentis* in larvae was longer than in E3 (2–3 days in larvae compared to 1 day in E3) (Figure 5D), suggesting some interaction of these bacteria within the host, such as adhering to the mucus or obtaining nutrients. The survival of *C. difficile* in E3 was similar to that observed in larvae inoculated at 3 dpf; bacterial counts were stable until 4 days post E3 inoculation. Because the E3 medium is mainly aerobic and incubated in aerobic conditions, it seems that more oxygen-resistant forms of *C. difficile* such as endospores could explain its survival in larvae and E3 medium. By contrast, when *C. difficile* was inoculated on 5 dpf larvae, it seems that it establishes a positive interaction with the host, since it could persist and proliferate.

### Endospores and vegetative cells persist in larvae

We then speculated that the persistence of *C. difficile* in zebrafish larvae could be due to the presence of endospores in larvae. To test this hypothesis we evaluated the concentration of vegetative cells and endospores of *C. difficile* in larvae. We compared these results to those for another sporulating bacterium, the aerobic probiotic *Bacillus clausii*, since *Bacillus* was one of the bacterial taxa from the human fecal microbiota which could persist in larvae (Figure 2E; Table 1). We observed that *B. clausii* can colonize the gastrointestinal tract of larvae, since fluorescent bacteria (Figure 3A, middle panel) were observed in this organ (Figure 4), similar to *C. difficile* (Figures 3B, 4). Both bacteria can form endospores (Figures 3A,B, lower panels) when they grow in agar medium (Figures 3A,B, upper panels).

**Figure 6:**
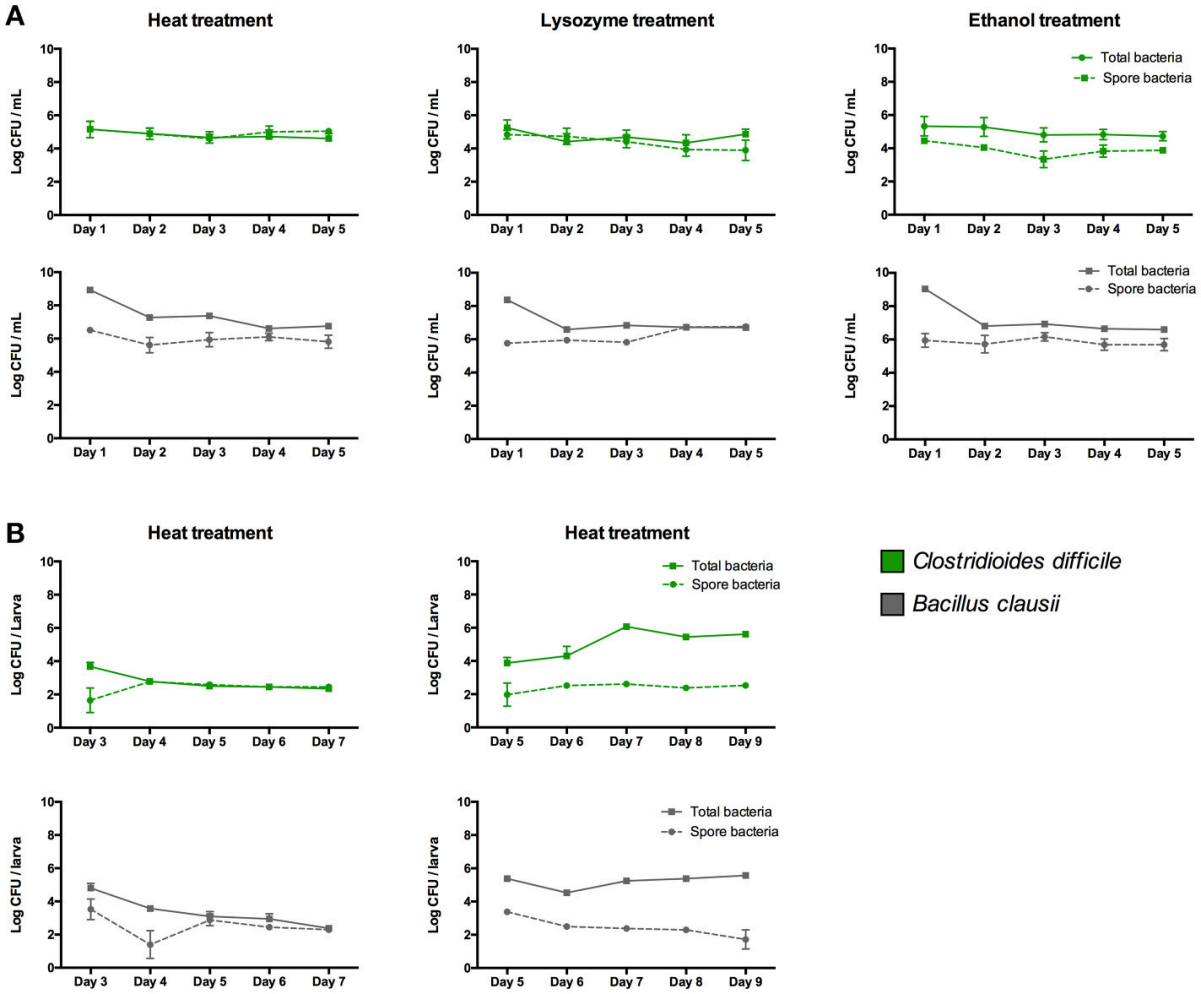
Concentration of total cells and endospores of *Clostridioides difficile* and *Bacillus clausii* when they are inoculated in **(A)** E3 medium and in **(B)** 3 or 5 dpf zebrafish larvae. Endospore concentrations were determined by treating samples at 75°C for 15 min, or lysozyme (2 mg/mL) for 1 h at 37°C, or 50% ethanol for 30 min at 37°C, before plating in NN or TSA medium. Each colonization and sporulation experiment was performed independently at least 3 times; the results show the mean ± SE.

When we inoculated the zebrafish media (E3), we observed that almost 100% of cells detected were heat, lysozyme or ethanol-resistant endospores (Figure 6A). In the case of *B. clausii*, endospores represented about 0.1% of the total cells the day of inoculation in zebrafish media, increasing to almost 100% at day 4 after inoculation. In colonization experiments endospores were detected only as heat resistant cells, since the 3 treatments showed similar results. Approximately 10% of the inoculated *C. difficile* and *B. clausii* cells at inoculation day in 3 dpf larvae were endospores (Figure 6B). This percentage increased to 100% at day 5 dpf for *B. clausii* and at day 4 dpf for *C. difficile* (Figure 6B). However, when both bacteria were inoculated in 5 dpf larvae, an important proportion (>98%) of the two bacteria were in the vegetative form until the end of the experiment (9 dpf) (Figure 6B). The vegetative count of *C. difficile* increased about 2 log, suggesting proliferation of the inoculated bacteria and better colonization of the host.

## Discussion

In this study, to humanize zebrafish larvae we used conventionally raised larvae harboring their own microbiota. These first colonizers would create a new environment that promotes the colonization of strict anaerobes, as observed in the early colonization of the human gut. In humans, the early gut colonization is characterized by a lack of stability, where the microbiota composition can be influenced by different temporal environmental factors such as birth mode, type of milk consumption and weaning (Laforest-Lapointe and Arrieta, 2017). The first microorganisms are facultative anaerobes such as Enterobacteriaceae (γ-Proteobacteria), *Staphylococcus* and *Streptococcus* (Firmicutes), which consume the available oxygen in the gut (Laforest-Lapointe and Arrieta, 2017). Early gut colonization of zebrafish larvae is characterized by a high abundance of facultative anaerobe γ-Proteobacteria, with a relative instability of the microbiota and important effect of the surrounding environment, similar to humans (Stephens et al., 2015). This instability could favor the establishment of allochthonous microorganisms as in our previous colonization of zebrafish larvae with probiotic yeasts (Caruffo et al., 2015, 2016). The results of this study showed that the larval microbiota was composed of aerobic bacteria of the Phylum Proteobacteria, as previously reported (Rawls et al., 2004; Stephens et al., 2015). Our study tested the effect of habitat on the assemblage of the transplanted microbiota, since larvae are not fed at this stage of zebrafish development. Therefore, the colonization capacity of the allochthonous microorganisms is determined by interactions with the host gut, interactions with the larval microbiota or by intrinsic bacterial factors.

In the first strategy, we inoculated a homogenized fecal sample from a human volunteer. This fecal microbiota was dominated by the phylum Bacteroidetes and Firmicutes, as previously described (Hugon et al., 2015; Fujio-Vejar et al., 2017). Then we homogenized the fecal sample by an experimental approach to capture a remarkable proportion of a person's fecal microbiota (Goodman et al., 2011). We checked that the supernatant of the homogenized sample, which was used as the larval inoculum, had a similar bacterial composition to the fecal sample, as previously reported (Goodman et al., 2011). Anaerobic culture analyses on M2GSC medium with rumen fluid (Barcenilla et al., 2000) and NN medium adequately described the genera identified by MiSeq sequencing and identified several fastidious bacteria. However, it is important to note that these microorganisms were identified from Petri plates containing confluent colonies (>1,000) and not in plates with isolated bacterial colonies (data not shown). This reveals the importance of bacterial interactions between microbiota organisms in their successful *in vivo* and *in vitro* growth. Similar to a previous report in human gut microbiota, the good recovery of the microbiota through culture could be attributed to syntrophic interactions between different microorganisms (Lau et al., 2016).

It has been shown that it is feasible to colonize zebrafish larvae with allochthonous mice microbiota. When germ-free zebrafish larvae (3 dpf) were transplanted with a pooled cecal content of adult mice by immersion in larval medium (Rawls et al., 2006), aerobic bacteria from the γ-Proteobacteria (*Escherichia/Shigella, Proteus*) and Bacilli (*Enterococcus, Staphylococcus*) colonized the larvae. These strains are aerobic and their colonization can be facilitated by a more aerobic intestine in this larval stage (Rawls et al., 2006; Stephens et al., 2015). In zebrafish, the colonization with a more anaerobic microbiota occurs at a late stage of development, in adulthood (Stephens et al., 2015), similar to humans (El Aidy et al., 2013). In our study, when 3 dpf larvae were inoculated with human gut microbiota a total of 6 bacterial taxa belonging to the phyla Bacteroidetes, Firmicutes and Proteobacteria were transplanted. Interestingly, 4 of these bacteria are strict anaerobes (*Prevotella* (Bacteroidetes), *Oscillospira* (Firmicutes), *Roseburia* (Firmicutes), and an unclassified Ruminococcaceae (Firmicutes) and frequently described as members of a healthy microbiota. *Roseburia* is a well characterized butyrate-producing bacterium (Tamanai-Shacoori et al., 2017). *Prevotella* has been described as a representative member of enterotype 2 (Arumugam et al., 2011) associated with long-term consumption of carbohydrate-rich diets. For the first time, we have cultured *Oscillospira* from a human fecal sample and detected it in transplanted larvae. *Oscillospira* is a frequent bacterium of the gut microbiota associated with health and leanness (Konikoff and Gophna, 2016). Because this bacterium is expected to grow in human gut mucins (Konikoff and Gophna, 2016), one could speculate that it also can utilize larval mucins. Two aerobic bacteria were also successfully transplanted: *Bacillus* (Firmicutes) and one unclassified taxon of the Enterobacteriaceae (Proteobacteria). *Bacillus* is an aerobic microorganism which has been identified as a low abundance but highly prevalent component of the human gut microbiota (Hoyles et al., 2012), and Enterobacteriaceae is a highly prevalent family. It is important to note, however that only *Bacillus* persisted in larvae and detected from 4 to 7 dpf larvae. Interestingly, it seems that the transplanted microorganisms stimulated 33 new taxa that were only identified in inoculated larvae. This could be explained by specific bacterial interactions, such as a syntrophic relationship between human and larval microbiota. Together, these results revealed that transplanting the entire human gut microbiota to conventionally raised larvae was a successful strategy to transfer some more anaerobic members of the human gut microbiota than previously described in germ-free larvae (Rawls et al., 2006). However, transplanted microorganisms did not persist in the larval gut, probably because they did not encounter their specific ecological niches or were not detected due to the low sensitivity of the methods used, limiting the utility of this model.

We then explored a second strategy consisting of inoculating aerotolerant anaerobic microorganisms, as a simulation of human gut colonization (El Aidy et al., 2013). The results revealed no positive interactions between these bacteria. *Lactobacillus* and *Bifidobacterium* did not persist in the gut of larvae more than 3 days after inoculation. This timeframe, however was longer than their survival in the larval medium and longer than the intestinal transit time (Cocchiaro and Rawls, 2013), suggesting some interaction within the larval gut, such as adhering to the mucus (Ouwerkerk and de Vos, 2013) or interacting with members of the larval microbiota, that potentially delayed their exit from the host.

The ability to adhere and grow in mucus has been regarded as one factor increasing the colonization capacity of a microorganism. Intestinal mucus is composed of mucin polymers or glycoproteins of sialic acid-capped O-glycan chains attached to the protein backbone via serine/threonine residues, which are considered substrates for the growth of some intestinal bacteria (van Passel et al., 2011; Tailford et al., 2015). In humans some mucins are attached to the epithelial membrane (e.g., MUC1 and MUC4) or secreted into the gut lumen (e.g., MUC2 and MUC5B) (Tailford et al., 2015). A specific glycosylation pattern which can vary through cell-specific glycosyltransferase expression levels can determine the type of bacteria residing in this niche. In zebrafish, the differentiation of mucus-producing intestinal goblet cells which are localized in the mid-intestine and production of MUC2.1 is evident at 100 h post fertilization (about 4 dpf) (Ng et al., 2005; Lai et al., 2016). There is no information about the glycosylation pattern of larval mucins, however we know that the first colonizers of the zebrafish can persist in the gut in the early stage of larval development in the absence of exogenous feeding by adhering to the mucus (Rawls et al., 2004; Bates et al., 2006) and probably by taking nutrients from it. *Lactobacillus* and *Bifidobacterium* can adhere to mucus through mucus-binding protein (Ouwerkerk and de Vos, 2013); they are expected to use host-produced carbohydrates such as human intestinal mucins in the absence of external feeding (Ouwerkerk and de Vos, 2013; Tailford et al., 2015; Rivière et al., 2016). However, they disappeared from the larval gut 3 days after being inoculated. This suggests that they cannot metabolize larval mucins or they could not reach this specific niche, e.g., due to the presence of the larval microbiota.

In contrast, *Clostridioides* was the most persistent in the larval gut, reaching the same density at the end of the experiment as on the inoculation day (3 dpf). Moreover, it could proliferate when it was inoculated on day 5 dpf, similar to the sporulating probiotic *B*. *clausii*. It is known that *C. difficile* forms endospores that facilitate its persistence within the human host and transmission through the environment (Browne et al., 2016). *B. clausii* endospores are also able to survive and persist in the human gut (Lopetuso et al., 2016). Thus we speculated that the persistence of these bacteria in larvae could be facilitated by this biological process. We determined the concentration of endospores in inoculated larvae and in E3 medium. For both bacteria we observed that an important proportion of bacterial cells were endospores when they were inoculated in 3 dpf larvae and E3. This suggests the importance of sporulation in the persistence of these microorganisms in the environment as well in the gastrointestinal tract, similar to a previous report in humans (Browne et al., 2016). Interestingly, when these two sporulating bacteria were inoculated in 5 dpf larvae, the concentration of vegetative cells increased for *C. difficile* and was stable for *B. clausii*, suggesting proliferation of vegetative cells or germination of the endospores. The differences observed in the colonization capacity when they were inoculated at 3 or 5 dpf could be due to differences in larval development. Endospore germination could occur in a more mature larval gut probably because of the presence of bile acids, which are known to stimulate endospore germination in human sporulating bacteria (Browne et al., 2016). Active development of the gut is occurring in this period in zebrafish larvae. Lumen formation initiates at 30–52 hpf in the rostral region and advances caudally, becoming a continuous lumen at 74–76 hpf (~3 days), but the anus remains closed until 98 hpf (~4 days), when intestine becomes a completely open-ended tube (Ng et al., 2005). Also, by the end of 4 dpf there is a massive expansion of the intestinal tract in the rostral region giving rise to the intestinal bulb, thus increasing gut capacity (Ng et al., 2005). However, more studies are needed to decipher specific zebrafish factors influencing the colonization capacity of the microorganisms. These results highlight the importance of the timing of a bacterial inoculation as well as sporulation as factors that can influence the colonization capacity of microorganisms.

It is important to note that our colonization experiments were performed with sporulating cultures, in which about 10% of the total cells were endospores (Figure 3, lower panels). However, to demonstrate clearly that sporulation is an essential trait to colonize zebrafish larvae, isogenic mutants unable to form spores (e.g., mutant in *spo0A* gene) should have been used to confirm this statement, as previously reported in mice (Deakin et al., 2012). It has been recently reported that at least 50–60% of bacterial genera representing a relative abundance of about 30% of the human gut microbiota are able to sporulate, explaining why a significant proportion of oxygen-sensitive bacteria can be transmitted between individuals (Browne et al., 2016). In this report, some of the bacterial species suspected of forming endospores belong to the genus *Eubacterium*. This could explain why in the study of Toh et al. (2013) the strict anaerobe *Eubacterium limosum* was successfully implanted into 5 dpf zebrafish larvae and detected 3 days post-inoculation. This sporulation phenotype could also explain the successful transplant of *Bacillus* from the entire human fecal microbiota to zebrafish larvae in our study, as well as the inoculation of more developed larvae (5 dpf).

In summary, conventionally raised larvae can support the inoculation of more human gut species than previously reported, however most of the bacteria from the human gut microbiota were unable to persist in larvae, suggesting that more studies are needed to develop a zebrafish model colonized with human gut microbiota. Inoculation of specific bacteria such as *C. difficile* and *Bacillus* showed that these bacteria were able to colonize and proliferate when they were inoculated in 5 dpf larvae, highlighting the importance of host factors, such as the developmental stage in the colonization capacity of microorganisms. However, to determine specific interactions with the host, future studies are needed using germ-free larvae. Colonizing germ-free larvae with early human gut aerobic microbiota such as *Escherichia coli, Enterococcus*, or *Staphylococcus*, etc. could help the colonization of anaerobic microorganisms such as *Prevotella, Bacteroides*, or even more fastidious anaerobes such as *Faecalibacterium prausnitzii* or *Akkermansia muciniphila*, among others.

In conclusion, our study highlights the utility of using zebrafish larvae to decipher some factors affecting colonization by human gut microorganisms. The results suggest that the developmental stage of larvae and bacterial sporulation may be factors that can affect the colonization ability of the strains. However, more studies are needed to confirm these observations. Finally, the use of germ-free larvae would aid to understand how some human gut microorganisms might interact with this host model.

## Data availability

The raw data paired-ends reads obtained from the MiSeq platform were stored in the ENA online public database with accession number PRJEB19263 (http://www.ebi.ac.uk/ena/data/view/PRJEB19263). The 16S rRNA amplicon sequencing data generated from bacterial isolated were deposited in the GenBank database under the following accession numbers: KY523549-KY523592.

## Author contributions

CF and SM raised the zebrafish larvae. M-JV, YH, and MaxC performed the colonization and sporulation experiments. MT and GF contributed with culture analysis. DM and DG performed the bioinformatics analysis. M-JV and MarC prepared the Figures. PN, M-JV, MarC, AR-J, MT and FM analyzed the results. PN and FM wrote the paper. All authors reviewed the manuscript. M-JV and MarC contributed equally to this work.

### Conflict of interest statement

The authors declare that the research was conducted in the absence of any commercial or financial relationships that could be construed as a potential conflict of interest.

## Supplementary material

Supplementary Video 1 DTAF-labeled *C. diffilice* located in the gastrointestinal tract of larvae.
